# Sensitivity of Calcification to Thermal Stress Varies among Genera of Massive Reef-Building Corals

**DOI:** 10.1371/journal.pone.0032859

**Published:** 2012-03-01

**Authors:** Juan P. Carricart-Ganivet, Nancy Cabanillas-Terán, Israel Cruz-Ortega, Paul Blanchon

**Affiliations:** 1 Unidad Académica de Sistemas Arrecifales, Instituto de Ciencias del Mar y Limnología, Universidad Nacional Autónoma de México, Puerto Morelos, Quintana Roo, México; 2 Unidad Chetumal, El Colegio de la Frontera Sur, Chetumal, Quintana Roo, México; University of Texas, United States of America

## Abstract

Reductions in calcification in reef-building corals occur when thermal conditions are suboptimal, but it is unclear how they vary between genera in response to the same thermal stress event. Using densitometry techniques, we investigate reductions in the calcification rate of massive *Porites* spp. from the Great Barrier Reef (GBR), and *P. astreoides*, *Montastraea faveolata*, and *M. franksi* from the Mesoamerican Barrier Reef (MBR), and correlate them to thermal stress associated with ocean warming. Results show that *Porites* spp. are more sensitive to increasing temperature than *Montastraea*, with calcification rates decreasing by 0.40 g cm^−2^ year^−1^ in *Porites* spp. and 0.12 g cm^−2^ year^−1^ in *Montastraea* spp. for each 1°C increase. Under similar warming trends, the predicted calcification rates at 2100 are close to zero in *Porites* spp. and reduced by 40% in *Montastraea* spp. However, these predictions do not account for ocean acidification. Although yearly mean aragonite saturation (Ω_ar_) at MBR sites has recently decreased, only *P. astreoides* at Chinchorro showed a reduction in calcification. In corals at the other sites calcification did not change, indicating there was no widespread effect of Ω_ar_ changes on coral calcification rate in the MBR. Even in the absence of ocean acidification, differential reductions in calcification between *Porites* spp. and *Montastraea* spp. associated with warming might be expected to have significant ecological repercussions. For instance, *Porites* spp. invest increased calcification in extension, and under warming scenarios it may reduce their ability to compete for space. As a consequence, shifts in taxonomic composition would be expected in Indo-Pacific reefs with uncertain repercussions for biodiversity. By contrast, *Montastraea* spp. use their increased calcification resources to construct denser skeletons. Reductions in calcification would therefore make them more susceptible to both physical and biological breakdown, seriously affecting ecosystem function in Atlantic reefs.

## Introduction

Skeletal calcification in scleractinian corals generates large amounts of calcium carbonate substrate and offsets the physical and biological erosion of reefs [1], [2]. Calcification is an energy-consuming physiological process, and maximum rates occur when environmental conditions are optimal for skeletal growth [3]–[6]. As a consequence, calcification rate imparts information about a coral's environmental history [7], [8]. Although there are several environmental variables which affect coral calcification rates, such as light [9], [10], carbonate saturation state [11], water turbidity [12], [13], wave exposure [14] and reproduction rate [15], temperature has been shown to be particularly important. For example, during the annual seasonal cycle, the calcification rate increases as temperature increases, until it reaches a maximum in midsummer, after which it declines as temperature decreases [4], [16]. This produces the density-banding pattern in massive corals (somewhat analogous to tree-rings) that was first observed by Kuntson and coworkers [17]. In addition, where reefs develop down a gradient in sea surface temperature (SST), the rate of coral calcification increases as SST increases [18], [19]. Lastly, short- and long-term experiments on corals adapted to a specific SST regime have shown that as temperature increases, coral calcification rate increases to a maximum and declines thereafter [20]–[23].

Reductions in calcification rates also occur when thermal conditions are suboptimal [24], and there have been several recent reports of a link between thermal stress and skeletal growth reductions in massive reef-building corals [25]–[30]. Such reports have mainly focused on the reconstruction of pre-Industrial SST, or on possible future scenarios for reduced coral skeletal growth due to ocean warming. But it is not yet clear how calcification rates vary between genera in response to the same thermal stress event. This question has important implications in light of future global warming scenarios because differential reduction in calcification between genera could potentially disrupt community structure, particularly if the affected genera are major reef-building species. Here we delineate the sensitivities of two major reef-building coral genera to thermal stress by examining recent historical variation in calcification rates in massive *Porites* from the Great Barrier Reef (GBR) and in massive *P. astreoides*, *Montastraea faveolata*, and *M. franksi* from the Mesoamerican Barrier Reef (MBR).

## Results

**Figure 1:**
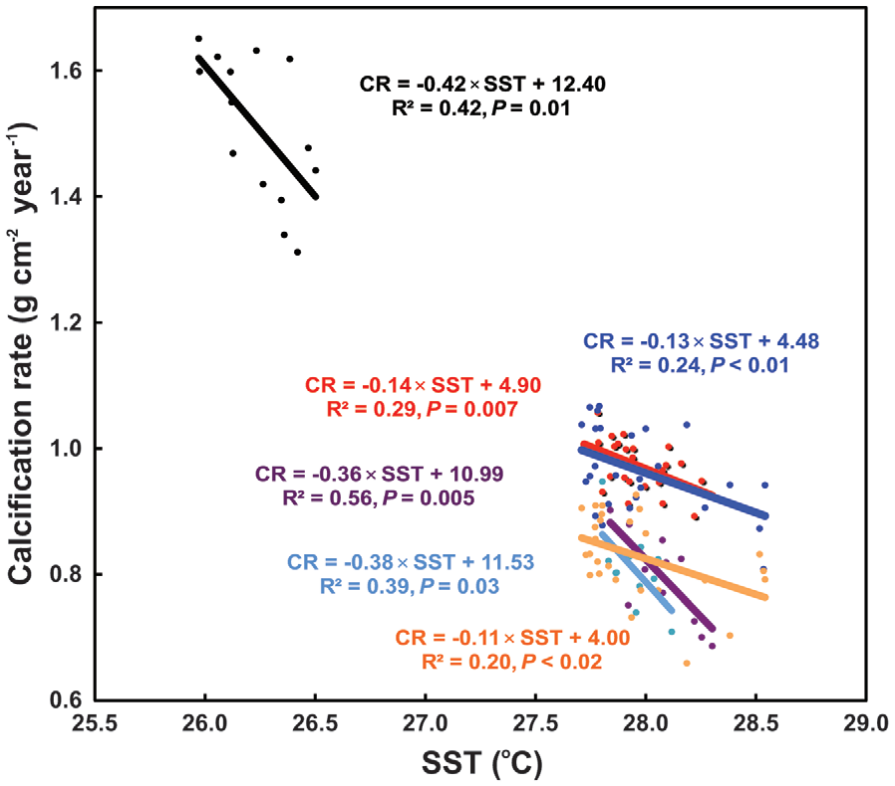
Mean annual calcification rates as a function of average annual sea surface temperature. In massive *Porites* spp. from Rib Reef, central Great Barrier Reef Australia (black), *Montastraea faveolata* from Mahahual (dark blue) and Chinchorro Bank, (red), Mesoamerican Barrier Reef System, *M. franksi* from Mahahual (orange), and *Porites astreoides* from Mahahual (light blue) and Chinchorro Bank (purple). CR = calcification rate, SST = sea surface temperature.

For all species in all reefs, calcification rate is negatively correlated with annual average SST (Fig. 1). In *Montastraea* spp. the calcification-rate slopes as a function of temperature are significantly lower than those of *Porites* spp. (*F*-test, *P*<0.05 in all cases). In addition, different species of *Porites* between the two regions show no significant differences in slope (*F*-test, *P*>0.05) suggesting this genus has a uniform response to thermal stress. The same is also true for *Montastraea* species in the MBR (*F*-test, *P*>0.05), although mean calcification rate in *M. franksi* was significantly lower in Mahahual (0.83 g cm^−2^ year^−1^) than in *M. faveolata* in Mahahual and Chinchorro Bank (0.96 g cm^−2^ year^−1^ and 0.97 g cm^−2^ year^−1^, respectively) (One-way ANOVA, Tukey's HSD, *P*<0.0001, *F* = 48.24). For *Porites* spp. the calcification rate decreases by 0.40 g cm^−2^ year^−1^ for each 1°C increase in temperature, whereas in *Montastraea* ssp. the decrease is only 0.12 g cm^−2^ year^−1^ (Fig. 1). Intercepts indicate calcification would cease at 30.0°C in *Porites* ssp., whereas for *Montastraea* spp. zero calcification is projected to occur at 35.0°C.

In Rib Reef, SST registered an increase trend of 0.4°C (R = 0.66, *P*<0.01), from 1989 to 2002, equivalent to 2.9°C per century. Over this 13-year interval, calcification rate in massive *Porites* spp. registered a reduced trend, decreasing around 20% (R = −0.76, *P*<0.001; Table 1). In the MBR, at Chinchorro Bank, SST also registered an increase of 0.6°C (R = 0.77, *P* = 0.0001), from 1985 to 2009, equivalent to 2.4°C per century. Over this 24-year interval, *M. faveolata* also registered a reduction of approximately 20% in calcification rate (R = −0.55, *P* = 0.001). By contrast, *P. astreoides* at Chinchorro suffered a 30% reduction in calcification (R = −75, *P* = 0.006) over a shorter 12-year interval, between 1998 and 2009 (Table 1). In Mahahual, however, no yearly SST trend was detected and mean calcification rates of *P. astreoides* and *Montastraea* species did not register a reduction during the analyzed time lines (1996 to 2006 and 1977 to 2003, respectively; Table 1).

**Table 1:** Correlation coefficients (CC) for sea surface temperatures (SST) as well as calcification rates for the coral species at the sampled reefs as a function of time (asterisks indicate significant correlations, *P*<0.05).

Sampled reef, SST and species	CC	Time line
**Rib Reef, Central Great Barrier Reef Australia**		
SST	0.66*	1989–2002
Calcification rate for massive *Porites*	−0.76*	1989–2002
**Mahahual Reef, Mesoamerican Barrier Reef System**		
SST	−0.20	1977–2006
Calcification rate for *Porites astreoides*	−0.51	1996–2006
Calcification rate for *Montastraea faveolata*	−0.14	1977–2003
Calcification rate for *Montastraea franksi*	0.35	1977–2003
**Chinchorro Bank, Mesoamerican Barrier Reef System**		
SST	0.77*	1985–2009
Calcification rate for *Porites astreoides*	−0.75*	1998–2009
Calcification rate for *Montastraea faveolata*	−0.55*	1985–2009

**Figure 2:**
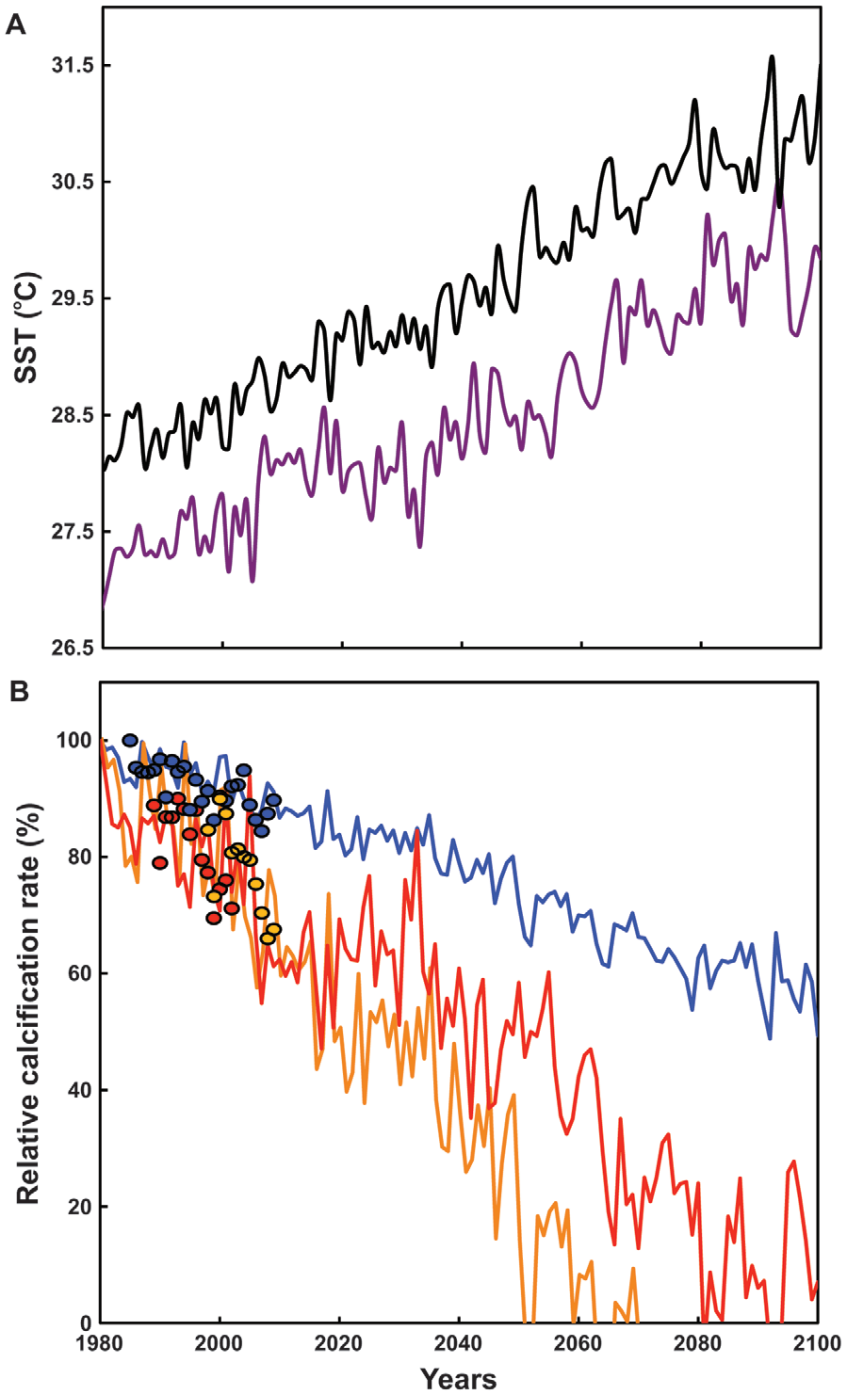
Modeled sea surface temperature and decreasing calcification rates for massive *Porites* spp. and *Montastraea* spp. from 1980 to 2100. (A) Modeled yearly mean sea surface temperature (SST) for the central Great Barrier Reef (purple line) and the Caribbean (black line) from 1980 to 2100. Modeled SST data are from Figures 10C and 8C in [32], respectively. (B) Modeled yearly mean relative calcification rate from 1980 to 2100 for massive *Porites* spp. (red line) in the Great Barrier Reef and *P. astreoides* (orange line), and *Montastraea* spp. (blue line) in the Mesoamerican Barrier Reef System. Yearly mean calcification rate data were generated with the regression lines of the relationship between calcification rate and SST (Figure 1) for massive *Porites* spp. growing in Rib Reef and *P. astreoides*, and *Montastraea faveolata* growing in Chinchorro Bank, using the modeled yearly mean SST presented in figure 2A. Red, orange and blue circles are the historical relative calcification rates of massive *Porites* spp. in Rib Reef and of *P. astreoides*, and *M. faveolata* in Chinchorro Bank, respectively.

Warming-model predictions of reduced calcification indicate that rates in massive *Porites* spp. from the GBR would be close to zero by 2100. Whereas, in the MBR, calcification rates in *P. astreoides* would be close to zero by 2060 and only be reduced around 40% by 2100 in *Montastraea* spp. (Fig. 2).

Around Mahahual and Chinchorro Bank yearly mean Ω_ar_ indicate a significant decrease from 2003 to 2010 (Fig. S1). *Porites astreoides* growing at Chinchorro Bank showed a significant increase of calcification rate associated with increasing Ω_ar_. In contrast, calcification rate in *M. faveolata* in Chinchorro Bank and *P. astreoides* in Mahahual showed no significant correlation with Ω_ar_ (Table S1).

## Discussion

Our comparison of the historical reduction in calcification rate between *Porites* spp. and *Montastraea* spp. to thermal stress during the three last decades, shows that *Porites* spp. are more sensitive to temperature increase than *Montastraea* spp. (Fig. 1). This differential sensitivity is clear at Chinchorro Bank, where calcification rate in *P. astreoides* is reduced 30% in comparison with *M. faveolata* (20%) in a 12-year shorter time interval. The reduction in calcification rate for massive *Porites* spp. in Rib Reef (20%, from 1989 to 2002) is similar to that reported by Cooper and coworkers [26] for this genus in two GBR inshore locations (21%, from 1988 to 2003). Later, De'ath and coworkers [27] also reported similar reductions for massive *Porites* spp. in several locations along the GBR. These authors suggested that the causes for this reduction are probably large-scale in extent and unprecedented within the past 400 years. By contrast, Lough and Barnes [31] reported a positive correlation between SST and calcification rate of massive *Porites* spp. growing in the GBR from 1906 to 1982. Thus, it is reasonable to presume that the negative impacts on calcification rate due to ocean-warming-induced thermal stress started in the 1980's on the GBR.

Although our analyzed time periods are too brief to exclude the effects of decadal-scale weather variability, the observed SST trends in Rib Reef and Chinchorro Bank are consistent with the warming predicted by most climate-change models [32], [33]. Associated with this warming, coral calcification rates in Rib Reef and Chinchorro Bank showed significant reductions (Table 1). Thermal sensitivity has been highlighted as the “Achilles' heel” of reef-building corals, and increases in SST above their upper thermal limit can have negative physiological consequences on energetic reserves [34] and tissue biomass [35]. The fact that in Mahahual, SST and calcification rate of *P. astreoides* and *Montastraea* spp. showed no tendency through time, and that calcification rates of these species were negatively correlated with SST, implies that in recent decades coral species there have been exposed to frequent, intense, but short-lived thermal stress events. For example, although thermal stress does not necessarily need to cause coral bleaching (i.e., whitening of corals due to loss of symbiotic algae and/or their pigments) in order to reduce calcification [25], short-lived reductions in calcification have been reported for several reef-building corals following thermal-induced bleaching events [15], [36]–[38]. Bleaching events are expected to occur when the current SST reaches 1°C over the maximum monthly mean SST [39], and in the last decades extensive bleaching events occurred along the MBR [40].

The higher sensitivity of *Porites* spp. calcification to temperature increase is reflected in the warming-model predictions of reduced calcification. *Porites* spp. in the GBR and *P. astreoides* in the MBR are projected to cease calcification at the end of the century, whereas calcification of *Montastraea* spp. in the MBR will be reduced by only 40%. (Fig. 2). It is worth mentioning that these predictions ignore coral mortality, and the negative effects on coral calcification rate caused by bleaching events and other stressors. Furthermore, massive *Porites* spp. and *Montastraea* spp. are major reef-building corals in the Indo-Pacific and Atlantic oceans [41]–[43], and differential reductions in calcification as a result of thermal stress associated with warming in these oceans, might be expected to have significant ecological repercussions. One specific example of this involves growth strategies: *Porites* spp. invest their energy in growing faster and reduced calcification therefore translates into a decrease in extension rate rather than a decrease in density [18], [44]. By contrast, *Montastraea* spp. very their skeletal density to maintain extension rate, and reductions in calcification therefore result in decreased skeletal density [7], [19]. Any reduction in the extension rate of *Porites* spp. may reduce their ability to compete for space within a reef, whereas reductions in density in *Montastraea* spp. would increase their susceptibility to both physical and biological breakdown.

Corals provide the primary framework of a reef [45], and this forms the structural basis of the large biological diversity associated with them [46]–[48]. Therefore, along with other differential stressors at the genus level, such as bleaching and disease [49], [50], the deleterious impact of ocean warming on the skeletal growth strategies of major reef-building corals could potentially disrupt community structure in both Indo-Pacific and Atlantic reef systems. In much of the Indo-Pacific, massive *Porites* spp. are common and a reduction in their ability to compete for space could easily be compensated for by a shift in taxonomic composition [51], although this might have uncertain repercussions for biodiversity. Further, in areas of reduced coral diversity, such as the east Pacific, where massive *Porites* spp. play a high significant ecological role [52], reductions in their calcification rate might have more serious repercussions. In the Atlantic the major reef-building genera are branching *Acropora* and massive *Montastraea*. As a consequence, particularly in light of the Caribbean-wide decline in *Acropora palmata* and *A. cervicornis* that began in the mid-1980's, and the flattening of reefs that followed [53]–[55], anything that impacts the calcification rate of *Montastraea* spp. could seriously affect ecosystem function. Moreover, *P. astreoides* is becoming increasingly dominant on Caribbean reefs [56], [57] and the rapid reduction of its calcification rate could have far more serious repercussions.

Finally, a reduction in aragonite saturation state (Ω_ar_), due to elevated *p*CO_2_ associated with global warming, has also been highlighted as a stressor that negatively affects coral calcification [58]–[60]. It has been shown recently that the calcification response to changing Ω_ar_ among individual coral species is highly variable and often nonlinear, and that there could be additional factors contributing to the variation in calcification between reefs that might offset and subsequently mask the effects of decreasing Ω_ar_ [60]. We were unable to explore such potential variation with our current Ω_ar_ data set due to limitations in Ω_ar_ resolution and accuracy prior to 2003. In addition, our calcification rate data corresponding to the usable Ω_ar_ data of 2003–2010 is not available for all species at all sites (see materials and methods). Nonetheless, our results so far suggest that there is no effect of changes on Ω_ar_ on coral calcification rate in the Mexican Caribbean. This is supported by the fact that, even where there is a historical decrease of Ω_ar_ around Mahahual and Chinchorro Bank, only *P. astreoides* growing at Chinchorro Bank showed a significant positive correlation with Ω_ar_. Furthermore, at Mahahual no species experienced historical reduction of calcification rates. However, future work is needed to determine if there is an additional effect of Ω_ar_ over and above that of temperature, in order to improve predictions of how reef ecosystems will respond to forecasted Ω_ar_ decreases.

## Materials and Methods

### Study sites

**Figure 3:**
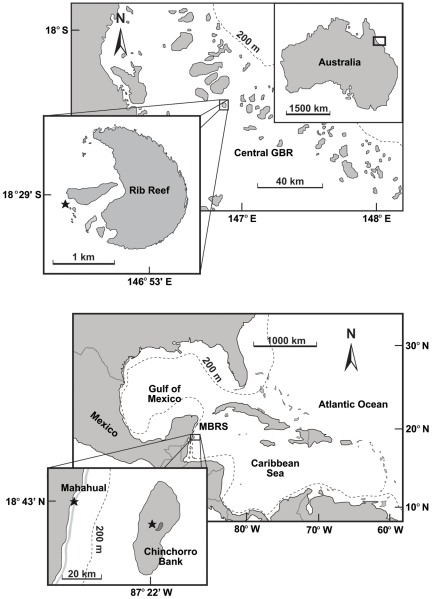
Location of reefs where corals were collected. Rib Reef, central Great Barrier Reef, and Mahahual and Chinchorro Bank, Mesoamerican Barrier Reef. The stars indicate where corals were collected on each reef location.

Samples were collected in three reef locations (Fig. 3): 1) Rib Reef, on the central GBR, Australia, is a 4 km^2^ mid-shelf reef located 56 km offshore (∼18° 29′S; 146° 53′E); 2) Mahahual Reef (∼18°43′N; 87°41′W), a fringing reef that occurs on the south-east coast of the Yucatán Peninsula; and 3) Chinchorro Bank (∼18°23′−18°41′N; ∼87°14′−87°27′W), an isolated platform, 48 km long and 18 km at its widest part, with a lagoon area >500 km2, located 27 km east of Mahahual, in the Mexican Caribbean. Both Mahahual and Chinchorro Bank form part of the MBR. The permits to collect the samples were provided in Australia by the Great Barrier Reef Marine Park Authority (GBRMPA), and in Mexico by the Secretaría de Agricultura, Ganadería, Desarrollo Rural, Pesca y Alimentación (SAGARPA).

### Coral collection

At Rib Reef, nine colonies of *Porites lutea*, two of *P. australensis* and one of *P. mayeri*, all between 110 and 210 mm in height and growing between 3- and 10 m depth, were collected in December 2002 [61]. Lough and coworkers [62] found that annual calcification rate of these three species is not statistically different. It was therefore considered reasonable to combine calcification rate data for these three species. At Mahahual Reef, seven colonies of *P. astreoides*, all ∼200 mm in height, were collected in September 2007; three cores of *Montastraea faveolata*, and three of *M. franksi* were collected in April 2006, all of them growing in ∼3 m of water. At Chinchorro Bank, four colonies of *P. astreoides*, all ∼200 mm in height, and eight cores of *M. faveolata*, were collected in March 2010: all living coral colonies were growing in ∼3 m of water. Colonies of massive *Porites* spp. from Rib Reef and *P. astreoides* from the two locations in the Mexican Caribbean were collected with hammer and chisel, and all *Montastraea* spp. cores were drilled along the main growth axis of the coral (i.e., one core drilled from one colony), by a diver using a rotary pneumatic hand drill fitted with a 3-cm-diameter, 38-cm-long diamond-bit core barrel.

### Calcification rate data

A rock saw equipped with a diamond-tipped blade was used to cut a ∼7-mm-thick axial slice from each coral colony and core. All slices were air-dried and X-radiographed. Bulk density series along the main growth axis were obtaining using direct gamma (Am^241^) densitometry of skeletal slices [63] for GBR massive *Porites* spp. [61], and densitometry from digitized images of X-radiographs [64] for *Montastraea* spp. and *P. astreoides*. In such density series (bulk density; g cm^−3^), extension rate (linear growth rate; cm year^−1^) was measured from successive density minima in all *Porites* specimens [61], [65], [66], and from successive density maxima in *Montastraea* specimens [16]. Then, in all specimens, annual calcification rate was calculated as the product of the annual extension rate and the average density of skeleton deposited in making that extension (gCaCO_3_ cm^−2^ year^−1^ = cm year^−1^ · gCaCO_3_ cm^−3^) [16]. Mean annual calcification rates were obtained by averaging annual values from each year, between colonies of the same species collected in the same reef location (Table S2).

### Sea surface temperature (SST)

Annual mean SSTs for each sampling locality were obtained from the Hadley Centre Sea Ice and SST (HadISST) data set produced by the United Kingdom Meteorological Office. These data are monthly averages of SST measurements taken from the Met Office Marine Data Bank (MDB), which also includes data received through the Global Telecommunications System (GTS) from 1982 onwards. In order to enhance data coverage where there are no MDB data, the HadISST data set uses monthly median SSTs for 1871 to 1995 available from the Comprehensive Ocean-Atmosphere Data Set (COADS) (see [67] for a more extensive discussion on HadISST data set precision and uncertainty).

### Aragonite saturation state (Ω_ar_)

Associated with Mahahual and Chinchorro Bank, yearly mean Ω_ar_ from 2003 to 2010, were calculated using the Ocean Acidification Product Suite (v0.5), produced by the National Oceanic and Atmospheric Administration (NOAA) Coral Reef Watch. The model runs nominally at 25 km resolution. Unfortunately, prior to November 2003 the model depends upon World Ocean Atlas salinity climatologies. As a result, the data prior to November 2003 are coarse and are associated with a substantial landmask [68].

### Calcification rates from 1980 to 2100

Yearly mean calcification rate data for massive *Porites* spp. from Rib Reef, GBR, and *P. astreoides* and *Montastraea* spp. from Chinchorro Bank, MBR, were correlated with modeled SST from 1980 to 2100 using a linear regression. Modeled yearly mean SST values for this period of time for the central GBR and the Caribbean were reported by Hoegh-Guldberg [32 (Figs. 10C and 8C, respectively)].

### Statistical analysis

A one-way ANOVA, followed by a Tukey's HSD, was used to examine the difference between calcification rates of *M. faeolata* growing in Chinchorro Bank and Mahahual, and *M. franksi* growing in Mahahual. To test for trends in time, linear regressions of annual SST and annual calcification rate of all species in all reef sites were calculated. Linear regressions of annual calcification rates of all species in all reef location versus SST were also calculated to examine the effects of thermal stress on calcification rate. The slopes of all linear regressions were compared with an *F*-test in order to look for different sensitivities between species and reef sites. Linear regressions were also used to test time trends in Ω_ar_ in the MBR and the effect of Ω_ar_ on the calcification rates of *M. faveolata*, *M. franksi*, and *P. astreoides* in Chinchorro Bank and Mahaual.

## Supporting Information

Figure S1 **Yearly mean aragonite saturation state (Ω_ar_), as a function of time (2003 to 2010), in Mahahual and Chinchorro Bank, Mesoamerican Barrier Reef.** Yearly mean Ω_ar_ were obtained using the Ocean Acidification Product Suite (v0.5), produced by the National Oceanic and Atmospheric Administration Coral Reef Watch (see Material and methods).
(TIF)

Table S1 **Correlation coefficients between aragonite saturation state (Ω_ar_) and calcification rate of** ***M. faveolata*** **and** ***Porites astreoides*** **growing in Chinchorro Bank and Mahahual, Mesoamerican Barrier Reef (asterisk indicate significant correlations,** ***P*** **= 0.01).**
(DOC)

Table S2 **Mean annual calcification rates and their standard deviation by reef location and species collected.** In parenthesis is the number of annual bands averaged in each case.
(DOC)
