# Supplementary figures and images for: Sensitivity of Calcification to Thermal Stress Varies among Genera of Massive Reef-Building Corals
Source: PLoS One. 2012 Mar 1;7(3):e32859. doi: 10.1371/journal.pone.0032859 (PMC3291612; doi:10.1371/journal.pone.0032859)

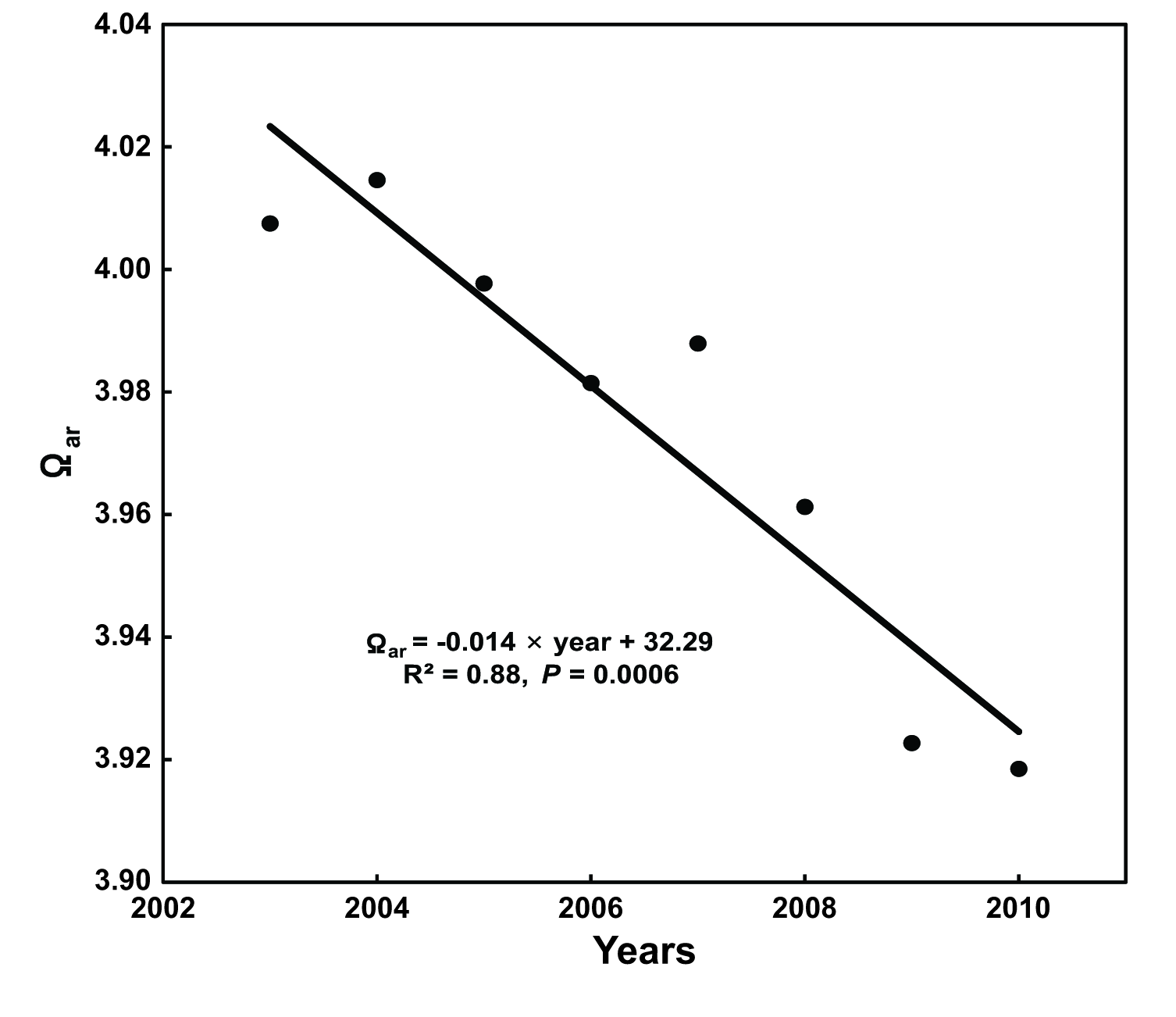

Supplement: Figure S1 — Yearly mean aragonite saturation state (Ωar), as a function of time (2003 to 2010), in Mahahual and Chinchorro Bank, Mesoamerican Barrier Reef. Yearly mean Ωar were obtained using the Ocean Acidification Product Suite (v0.5), produced by the National Oceanic and Atmospheric Administration Coral Reef Watch (see Material and methods). (TIF) [file pone.0032859.s001.tif]
