# Supplementary material for: Sensitivity of Calcification to Thermal Stress Varies among Genera of Massive Reef-Building Corals
Source: PLoS One. 2012 Mar 1;7(3):e32859. doi: 10.1371/journal.pone.0032859 (PMC3291612; doi:10.1371/journal.pone.0032859)
Supplement: Table S1 — Correlation coefficients between aragonite saturation state (Ωar) and calcification rate of M. faveolata and Porites astreoides growing in Chinchorro Bank and Mahahual, Mesoamerican Barrier Reef (asterisk indicate significant correlations, P = 0.01). (DOC) [file pone.0032859.s002.doc]

| Table S1. Correlation coefficients between aragonite saturation state (Ω_ar_) and calcification rate of *M. faveolata* and *Porites astreoides* growing at 3 m depth in Chinchorro Bank and Mahahual (asterisk indicate significant correlation, *P* = 0.01). | | |
| --- | --- | --- |
|  |  |  |
|  |  |  |
|  |  |  |
| Species and sampled reefs and depth | Correlation coefficients | Time line |
|  |  |  |
|  |  |  |
|  |  |  |
| *M. faveolata*, Chinchorro Bank (3 m depth) | 0.36 | 2003-2009 |
| *P. astreoides*, Chinchorro Bank (3 m depth) | 0.83* | 2003-2009 |
| *P. astreoides*, Mahahual (3 m depth) | 0.82 | 2003-2006 |
