# Supplementary material for: Sensitivity of Calcification to Thermal Stress Varies among Genera of Massive Reef-Building Corals
Source: PLoS One. 2012 Mar 1;7(3):e32859. doi: 10.1371/journal.pone.0032859 (PMC3291612; doi:10.1371/journal.pone.0032859)
Supplement: Table S2 — Mean annual calcification rates and their standard deviation by reef location and species collected. In parenthesis is the number of annual bands averaged in each case. (DOC) [file pone.0032859.s003.doc]

| **Table S2.** Mean annual calcification rates and their standard deviation by reef location and species collected. In parenthesis is the number of annual bands averaged in each case. | | | | | | |
| --- | --- | --- | --- | --- | --- | --- |
|  |  |  |  |  |  |  |
|  |  |  |  |  |  |  |
|  |  |  |  |  |  |  |
|  | **Sampled reefs and species** | | | | | |
|  |  |  |  |  |  |  |
|  | **Rib Reef** | **Mahahual Reef** | | | **Chinchorro Bank** | |
|  |  |  |  |  |  |  |
| **Year** | ***Porites* spp.** | ***M. faveolata*** | ***M. franksi*** | ***P. astreoides*** | ***M. faveolata*** | ***P. astreoides*** |
|  |  |  |  |  |  |  |
|  |  |  |  |  |  |  |
|  |  |  |  |  |  |  |
| 2009 |  |  |  |  | 0.95 ± 0.18 (8) | 0.70 ± 0.08 (4) |
| 2008 |  |  |  |  | 0.92 ± 0.26 (8) | 0.69 ± 0.11 (4) |
| 2007 |  |  |  |  | 0.89 ± 0.19 (8) | 0.73 ± 0.17 (4) |
| 2006 |  |  |  | 0.71 ± 0.32 (7) | 0.91 ± 0.16 (8) | 0.77 ± 0.05 (4) |
| 2005 |  | 0.97 ± 0.34 (3) | 0.95 ± 0.13 (3) | 0.80 ± 0.21 (7) | 0.94 ± 0.22 (8) | 0.81 ± 0.15 (4) |
| 2004 |  | 0.95 ± 0.21 (3) | 0.92 ± 0.15 (3) | 0.79 ± 0.37 (4) | 1.00 ± 0.16 (8) | 0.81 ± 0.16 (4) |
| 2003 |  | 0.97 ± 0.15 (3) | 0.77 ± 0.05 (3) | 0.82 ± 0.20 (3) | 0.98 ± 0.20 (8) | 0.82 ± 0.04 (4) |
| 2002 | 1.34 ± 0.41 (12) | 0.94 ± 0.22 (3) | 0.77 ± 0.07 (3) | 0.84 ± 0.08 (3) | 0.97 ± 0.25 (8) | 0.82 ± 0.11 (4) |
| 2001 | 1.42 ± 0.65 (12) | 0.96 ± 0.06 (3) | 0.79 ± 0.02 (3) | 0.80 ± 0.13 (3) | 0.95 ± 0.20 (8) | 0.88 ± 0.12 (4) |
| 2000 | 1.39 ± 0.41 (12) | 0.91 ± 0.33 (3) | 0.81 ± 0.30 (3) | 0.82 ± 0.09 (3) | 0.96 ± 0.27 (8) | 0.90 ± 0.24 (3) |
| 1999 | 1.31 ± 0.40 (12) | 0.95 ± 0.13 (3) | 0.90 ± 0.15 (3) | 0.78 ± 0.09 (3) | 0.91 ± 0.25 (8) | 0.75 ± 0.05 (2) |
| 1998 | 1.44 ± 0.61 (12) | 0.92 ± 0.24 (3) | 0.93 ± 0.32 (3) | 0.74 ± 0.08 (2) | 0.97 ± 0.44 (8) | 0.85 ± 0.12 (2) |
| 1997 | 1.48 ± 0.72 (12) | 0.91 ± 0.13 (3) | 0.88 ± 0.08 (3) | 0.83 ± 0.11 (2) | 0.95 ± 0.28 (8) |  |
| 1996 | 1.62 ± 0.63 (12) | 0.88 ± 0.27 (3) | 0.91 ± 0.05 (3) | 0.95 ± 0.55 (2) | 0.99 ± 0.32 (8) |  |
| 1995 | 1.55 ± 0.69 (12) | 0.89 ± 0.02 (3) | 0.86 ± 0.10 (3) |  | 0.93 ± 0.27 (8) |  |
| 1994 | 1.62 ± 0.40 (12) | 0.95 ± 0.14 (3) | 0.83 ± 0.04 (3) |  | 1.01 ± 0.32 (8) |  |
| 1993 | 1.65 ± 0.66 (10) | 1.07 ± 0.09 (3) | 0.83 ± 0.23 (3) |  | 1.00 ± 0.34 (8) |  |
| 1992 | 1.60 ± 0.84 (10) | 1.06 ± 0.27 (3) | 0.82 ± 0.24 (3) |  | 1.02 ± 0.21 (8) |  |
| 1991 | 1.60 ± 0.46 (10) | 1.07 ± 0.12 (3) | 0.80 ± 0.02 (3) |  | 0.95 ± 0.23 (8) |  |
| 1990 | 1.47 ± 0.31 (6) | 0.96 ± 0.04 (3) | 0.80 ± 0.21 (3) |  | 1.02 ± 0.19 (8) |  |
| 1989 | 1.63 ± 0.57 (3) | 0.97 ± 0.13 (3) | 0.88 ± 0.09 (3) |  | 1.00 ± 0.45 (8) |  |
| 1988 |  | 0.99 ± 0.13 (3) | 0.90 ± 0.24 (3) |  | 1.00 ± 0.29 (8) |  |
| 1987 |  | 1.03 ± 0.22 (3) | 0.89 ± 0.06 (3) |  | 1.00 ± 0.36 (8) |  |
| 1986 |  | 1.04 ± 0.12 (3) | 0.91 ± 0.41 (3) |  | 1.01 ± 0.19 (8) |  |
| 1985 |  | 1.03 ± 0.21 (3) | 0.91 ± 0.03 (3) |  | 1.06 ± 0.39 (8) |  |
| 1984 |  | 1.03 ± 0.13 (2) | 0.87 ± 0.04 (3) |  |  |  |
| 1983 |  | 0.91 ± 0.00 (2) | 0.79 ± 0.03 (3) |  |  |  |
| 1982 |  | 0.87 ± 0.33 (2) | 0.83 ± 0.17 (3) |  |  |  |
| 1981 |  | 0.81 ± 0.01 (2) | 0.81 ± 0.14 (3) |  |  |  |
| 1980 |  | 0.94 ± 0.03 (2) | 0.79 ± 0.20 (2) |  |  |  |
| 1979 |  | 0.94 ± 0.17 (2) | 0.70 ± 0.03 (2) |  |  |  |
| 1978 |  | 1.04 ± 0.04 (2) | 0.66 ± 0.17 (2) |  |  |  |
| 1977 |  | 1.02 ± 0.22 (2) | 0.73 ± 0.21 (2) |  |  |  |
|  |  |  |  |  |  |  |
